# Genomic characteristics of *cfr* and *fexA* carrying *Staphylococcus aureus* isolated from pig carcasses in Korea

**DOI:** 10.1186/s13567-024-01278-x

**Published:** 2024-02-16

**Authors:** Eiseul Kim, Seung-Min Yang, Hyo-Sun Kwak, Bo-Youn Moon, Suk-Kyung Lim, Hae-Yeong Kim

**Affiliations:** 1 Institute of Life Sciences & Resources and Department of Food Science and Biotechnology, Kyung Hee University, Yongin, 17104 Republic of Korea; 2 Bacterial Disease Division, Animal and Plant Quarantine Agency, Gimcheon, 39660 Republic of Korea

**Keywords:** *Staphylococcus aureus*, linezolid resistance, *Cfr* gene, *fexA* gene, whole-genome sequencing

## Abstract

The emergence of transferable linezolid resistance genes poses significant challenges to public health, as it does not only confer linezolid resistance but also reduces susceptibility to florfenicol, which is widely used in the veterinary field. This study evaluated the genetic characteristics of linezolid-resistant *Staphylococcus aureus* strains isolated from pig carcasses and further clarified potential resistance and virulence mechanisms in a newly identified sequence type. Of more than 2500 strains isolated in a prior study, 15 isolated from pig carcasses exhibited linezolid resistance (minimum inhibitory concentration ≥ 8 mg/L). The strains were characterized in detail by genomic analysis. Linezolid-resistant *S. aureus* strains exhibited a high degree of genetic lineage diversity, with one strain (LNZ_R_SAU_64) belonging to ST8004, which has not been reported previously. The 15 strains carried a total of 21 antibiotic resistance genes, and five carried *mecA* associated with methicillin resistance. All strains harbored *cfr* and *fexA*, which mediate resistance to linezolid, phenicol, and other antibiotics. Moreover, the strains carried enterotoxin gene clusters, including the hemolysin, leukotoxin, and protease genes, which are associated with humans or livestock. Some genes were predicted to be carried in plasmids or flanked by *ISSau9* and the transposon *Tn554*, thus being transmittable between staphylococci. Strains carrying the plasmid replicon *repUS5* displayed high sequence similarity (99%) to the previously reported strain pSA737 in human clinical samples in the United States. The results illustrate the need for continuous monitoring of the prevalence and transmission of linezolid-resistant *S. aureus* isolated from animals and their products.

**Supplementary Information:**

The online version contains supplementary material available at 10.1186/s13567-024-01278-x.

## Introduction

Staphylococci are common colonizers of the skin and mucous membranes of humans and animals and opportunistic pathogens in humans [1]. *Staphylococcus aureus* causes endocarditis, septicemia, pneumonia, abscesses, and meningitis. The combination of toxin-mediated virulence and antibiotic resistance complicates the treatment of *S. aureus* infection. Additionally, methicillin-resistant *S. aureus* (MRSA), which has limited treatment options, has spread globally [2]. Its intrinsic resistance to several common antibiotics and ability to acquire new antibiotic resistance genes increase treatment costs and the risk of treatment failure [3]. *S. aureus* also inhabits food-producing animals and livestock manure. *S*. *aureus*-related antibiotic resistance genes can be transferred from food-producing animals to humans, posing a significant potential risk to public health [4].

Linezolid, an oxazolidinone antibiotic, is the antibiotic of last resort for treating clinical infections caused by multidrug-resistant (MDR) Gram-positive bacteria, including MRSA, penicillin-resistant *Streptococcus pneumoniae*, and vancomycin-resistant *Enterococcus* species [5]. Linezolid resistance is mediated by chromosomal mutations in the V domain of 23S ribosomal RNA (rRNA), mainly G2576T/G2505A, or mutations in the L3/L4 ribosomal proteins [6]. In addition, linezolid resistance occurs through the acquisition of transferable resistance determinants (*optrA*, *poxtA*, and the chloramphenicol–florfenicol resistance gene [*cfr*]) via mobile genetic elements [7]. *cfr* encodes a methyltransferase that modifies 23S rRNA, spreads through plasmids, and confers cross-resistance to phenicols, lincosamides, oxazolidinones, pleuromutilins, and streptogramin A antibiotics [8]. *fexA* confers resistance to chloramphenicol and florfenicol. The presence of *fexA* in mobile genetic elements might contribute to the persistence of linezolid resistance [9].

In addition to antibiotic resistance, *S*. *aureus* carries several virulence factors [10] granting it the ability to evade, invade, and penetrate host immune defenses. Toxins commonly secreted by *S*. *aureus* include enterotoxin, leukotoxin, hemolysin, and toxic shock syndrome toxin-1 [11], in addition to surface proteins and enzymes. The secretion of enzymes (staphylokinase, coagulase, and lipase) and surface proteins (fibronectin proteins, collagen adhesin, and protein A) allows bacteria to evade host defenses and facilitates bacterial attachment, host tissue invasion, and penetration [12]. Most of these virulence factors act by degrading host molecules or interfering with metabolic pathways in the host [13].

Recent studies isolated linezolid-resistant *S. aureus* from humans, livestock, and food [14, 15]. Although the prevalence of linezolid resistance is low compared to that of other antibiotics, studies on transferable linezolid resistance genes and their virulence factors are ongoing [16]. These genes are commonly embedded in mobile genetic elements, such as plasmids and phages, or present as composite transposons in the bacterial chromosome [17, 18]. Mobile genetic elements allow the rapid distribution of linezolid resistance genes and virulence factors throughout the bacterial population. The acquisition of linezolid resistance and virulence genes by *S. aureus* from animals can lead to difficult to treat infections in livestock workers [14]. Therefore, it is important to understand the distribution of linezolid resistance and virulence genes and monitor *S. aureus* strains from food and livestock.

Whole-genome sequencing (WGS) can rapidly and consistently predict multiple genes and mutations associated with antibiotic resistance and simultaneously provide surveillance data [19]. This technique can additionally reveal phylogenetic relationships among pathogenic bacterial species, and typing methods based on genome sequence data are suitable for tracking foodborne outbreaks [14]. Phenotyping complements genotyping in making treatment decisions, as WGS primarily offers predictive insights. Moreover, depending on the sequencing technology used, it can be faster to determine the minimum inhibitory concentration (MIC). However, WGS can detect various antibiotic resistance gene mutations and functional alterations, providing deeper insights into the mechanisms of antibiotic resistance [20]. The present study analyzed the genetic properties of *cfr* and *fexA*-carrying *S. aureus* isolated from pig carcasses by genome sequencing and evaluated the phylogenetic relationships among the strains. Furthermore, we aimed to provide basic data on the interactions between *S*. *aureus* and its hosts by analyzing the virulence factors in strains isolated from pig carcasses in Korea.

## Materials and methods

### Collection of linezolid-resistant *S. aureus*

In our previous study, 2547 strains were isolated from animal carcasses (382 cattle, 1077 pig, and 1088 chicken carcass isolates) [21]. In our previous study, we isolated strains and determined their antibiotic susceptibility and resistance genes [21]. The isolates were identified using 16S rRNA gene sequences and matrix-assisted laser desorption/ionization time-of-flight mass spectrometry (BioMerieux, Marcy-l’Étoile, France). Their susceptibility to linezolid was measured using linezolid-containing plates (1–8 µg/mL) (Trek Diagnostic System Inc). Antimicrobial susceptibility of linezolid-resistant isolates was confirmed against 19 antimicrobial agents (cefoxitin, chloramphenicol, ciprofloxacin, clindamycin, erythromycin, fusidic acid, gentamicin, kanamycin, mupirocin, penicillin, quinupristin/dalfopristin, rifampin, streptomycin, sulfamethoxazole, tetracycline, tiamulin, trimethoprim, and vancomycin) using antimicrobial containing plates (Trek Diagnostic System Inc., Cleveland, Ohio, USA). The linezolid resistant strains were confirmed by polymerase chain reaction (PCR) targeting *cfr*, *fexA*, *optrA*, and *poxtA* genes. Methicillin resistance *S. aureus* was confirmed by PCR targeting *clfA* and *mecA* genes, and all strains identified as methicillin-resistant in the MIC test possessed the *mecA* gene [22].

### Whole-genome sequencing

Fifteen *S. aureus* strains harboring the *cfr* gene isolated in our previous study were cultured in tryptone soy broth at 37 ℃ for 24 h. For genomic DNA extraction, cultured strains were centrifuged at 16 000 × *g* for 15 min, and the pellets were washed with phosphate-buffered saline. After adding lysis buffer to the pellet, genomic DNA was extracted using the DNeasy Blood & Tissue kit (Qiagen, Hilden, Germany) according to the manufacturer’s instructions. The quality and concentration of DNA were determined using a MaestroNano micro-volume spectrophotometer (Maestrogen, Hsinchu, Taiwan).

For draft genome sequencing, libraries were constructed using the Illumina TruSeq DNA library prep kit (Illumina, San Diego, CA, USA). Genome sequencing was performed on a 300 bp paired-end Illumina MiSeq platform according to the manufacturer’s protocol. Raw data from Illumina MiSeq were cleaned by removing low-quality reads and adaptors and trimmed using Sickle version 1.33 default parameters. High-quality reads were assembled using SPAdes version 3.12. Assembled contigs were annotated using the Prokaryotic Genome Annotation Pipeline algorithm [National Center for Biotechnology Information (NCBI), Bethesda, MD, USA]. Draft genome sequences were deposited at GenBank [23] under accession numbers JAUPAV01 (LNZ_R_SAU_10), JAUPAW01 (LNZ_R_SAU_21), JAUPAX01 (LNZ_R_SAU_22), JAUPAY01 (LNZ_R_SAU_23), JAUPAZ01 (LNZ_R_SAU_24), JAUPBA01 (LNZ_R_SAU_25), JAUPBB01 (LNZ_R_SAU_26), JAUPBC01 (LNZ_R_SAU_27), JAUPBD01 (LNZ_R_SAU_31), JAUPBE01 (LNZ_R_SAU_37), JAUPBF01 (LNZ_R_SAU_46), JAUPBG01 (LNZ_R_SAU_57), JAUPBH01 (LNZ_R_SAU_58), JAUPBI01 (LNZ_R_SAU_62), and JAUPBJ01 (LNZ_R_SAU_64).

### In-silico typing

For identification, the average nucleotide identity (ANI) of each assembled genome against the genome of type strain (*S. aureus* DSM 20231^T^) was calculated using the ANI calculator [24]. ANI values of > 95% were considered the same bacterial species [24]. The nucleotide sequences of the assembled genomes were used as an input file, and the nucleotide sequence of the type strain was obtained from NCBI.

### Phylogenetic analysis

A total of 111 genome sequences isolated from pig sources were obtained from the NCBI to analysis comparative genomics (Additional file 1). This section was created by investigating high-quality and taxonomically accurate *Staphylococcus* genomes in the assembly database. Only genomes isolated from pig sources (swine facility, pig carcass, pork meat, and retail pork) were included. Pangenome analysis of 126 genomes (111 genomes obtained from the NCBI and 15 genomes sequenced in this study) was performed using Roary version 3.11.2. The phylogenetic tree was visualized using the Interactive Tree of Life program [25] based on the presence/absence of core and accessory genes. The multilocus sequence type (MLST) of 126 *S. aureus* genomes was identified using the PubMLST database (accessed on Jan 2, 2023).

### Detection of antibiotic resistance and virulence genes

Detection of antibiotic resistance genes in 15 genomes was performed using ResFinder version 4.1 with default parameters. Virulence genes were identified using VirulenceFinder version 2.0.3 with default parameters. The heatmaps for the presence/absence matrix of antibiotic resistance and virulence genes in these strains were visualized using the ggplot2 package of RStudio version 4.3.1.

### Mobile genetic elements

Mobile genetic elements in the 15 strains were detected using MobileElementFinder version 1.0.3. Insertion sequences were identified using ISfinder (accessed on Jan 2, 2023). Genomic islands were detected using IslandViewer 4 (accessed on July 27, 2023). The plasmid replicon was identified using PlasmidFinder version 2.1 default parameters (95% threshold for minimum % identity and 60% select minimum % coverage). Plasmids were verified using BLASTn, aligning assemblies to plasmid sequences of the NCBI RefSeq genome database (e-value 1e-5, identity > 95%, query coverage > 80%).

## Results

### Genomic features

In our previous study, 15 *S. aureus* strains harboring *cfr* were isolated from pig carcasses [21]. The assembly of the 15 sequenced strains harboring *cfr* revealed an average genome size of approximately 2 825 094 bp and an average G + C content of 32.5%. The number of scaffolds was ≤ 80, and the coverage was > 98 × . Information on the assembled genome characteristics is summarized in Additional file 1. The genomes of all strains included 2621–2734 coding genes. The assembled genomes were identified at the species level by analyzing average nucleotide identity, and the strains exhibited 97.3–98.93% identity with *S. aureus* DSM 20231^T^.

### Phylogenetic analysis of *S. aureus*

**Fig. 1:**
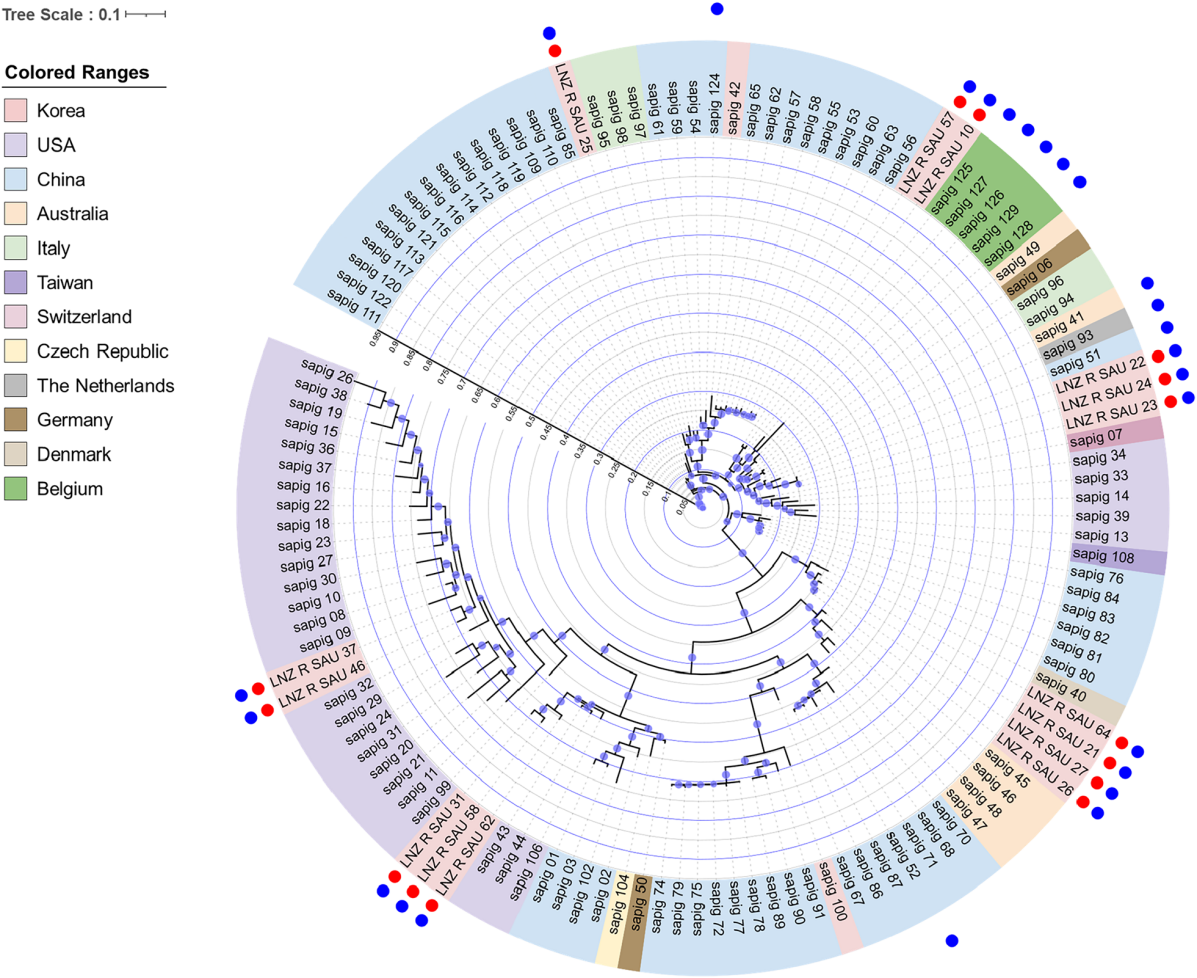
Phylogenetic tree based on presence/absence of core and accessory genes for 111 publicly available genomes of *S*. *aureus* isolated from pig-related sources and 15 genomes sequenced in this study. The colored range represents the country from which the strains were isolated. Internal tree scale and axis are displayed within the tree. Locations of genomes sequenced in this study are shown in the red circle. Strains carrying the *cfr* gene are indicated by blue circles.

To investigate the potential sources of strains, phylogenetic analysis was performed according to the presence and absence of accessory genes for 111 publicly available genomes of *S. aureus* isolated from pig-related sources and 15 genomes sequenced in this study (Fig. 1). Phylogenetic analysis revealed that the LNZ_R_SAU_37 and LNZ_R_SAU_46 strains were most closely related to *S. aureus* ISU 998 isolated in the United States (GCA_002274235.1). The LNZ_R_SAU_31, LNZ_R_SAU_58, and LNZ_R_SAU_62 strains were closely related to *S. aureus* NRRL B-41012 isolated in the United States (GCA_005153985.1). The LNZ_R_SAU_21, LNZ_R_SAU_26, LNZ_R_SAU_27, and LNZ_R_SAU_64 strains were closely related to *S. aureus* CFSAN018749 isolated in Denmark (GCA_003030065.1). The LNZ_R_SAU_25 strain was closely related to *S. aureus* GDB9P195A isolated in China (GCA_024916795.1). The LNZ_R_SAU_10, LNZ_R_SAU_22, LNZ_R_SAU_23, LNZ_R_SAU_24, and LNZ_R_SAU_57 strains were most closely related to *S. aureus* S681 isolated in Switzerland (GCA_002204735.1).

### Multilocus sequence typing (MLST) analysis

**Fig. 2:**
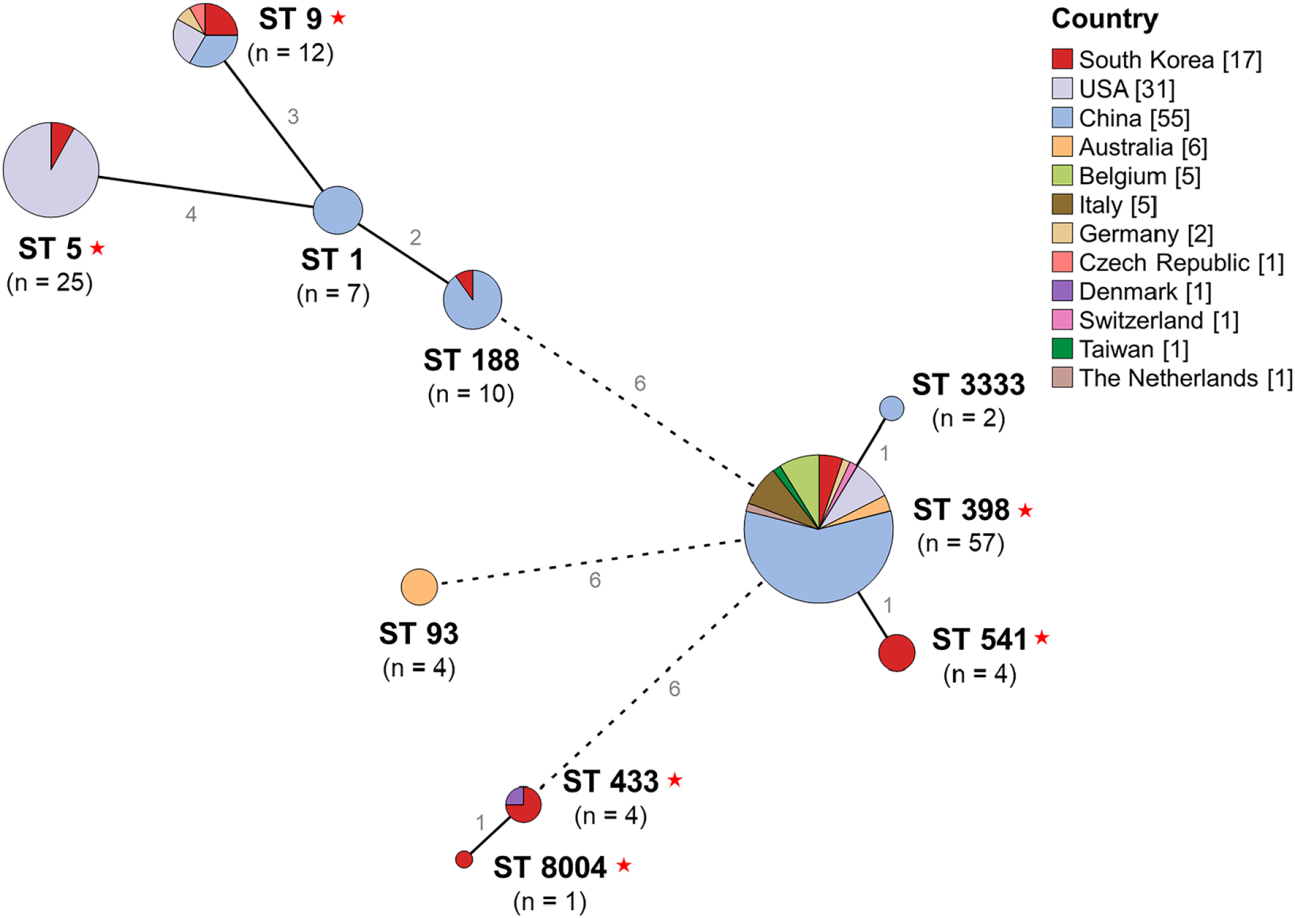
Minimum spanning tree by multilocus sequence type of *S*. *aureus* isolated from pigs or pig-associated environments. Each node within the tree represents a single sequence type. Node size is proportional to the number of genomes with that sequence type. Node color represents the country from which the strains were isolated. The branch length between each node indicates the number of allelic differences between the linked sequence types. Star indicates the sequence type related to the 15 isolates from this study.

The sequence types of the 15 strains sequenced in this study were ST398, ST541, ST433, ST9, ST5, and ST8004 (Additional file 2). The most frequent type was ST541 (26.7%; *n* = 4), followed by ST433 (20%; *n* = 3) and ST9 (20%; *n* = 3). The LNZ_R_SAU_64 isolate was identified with novel alleles and assigned a novel ID (ST8004) by the PubMLST team for curation and maintenance in the Bacterial Isolate Genome Sequence Database. A minimum spanning tree was generated by MLST analysis with the 15 strains sequenced in this study and other *S. aureus* strains of pig origin to evaluate phylogenetic relationships between strains isolated in various countries. MLST revealed the high diversity of *S. aureus* with distantly related strains (Fig. 2). The sequence types of 126 strains (111 publicly available genomes and 15 genomes sequenced in this study) were confirmed as ST398, ST5, ST9, ST188, ST1, ST541, ST93, ST433, ST3333, and ST8004. The most frequent type was ST398 (43.0%; *n* = 52), followed by ST5 (20.7%; *n* = 25) and ST9 (9.9%; *n* = 12). ST541 and ST8004 were identified only in the strains sequenced in this study.

### Detection of virulence factors

**Fig. 3:**
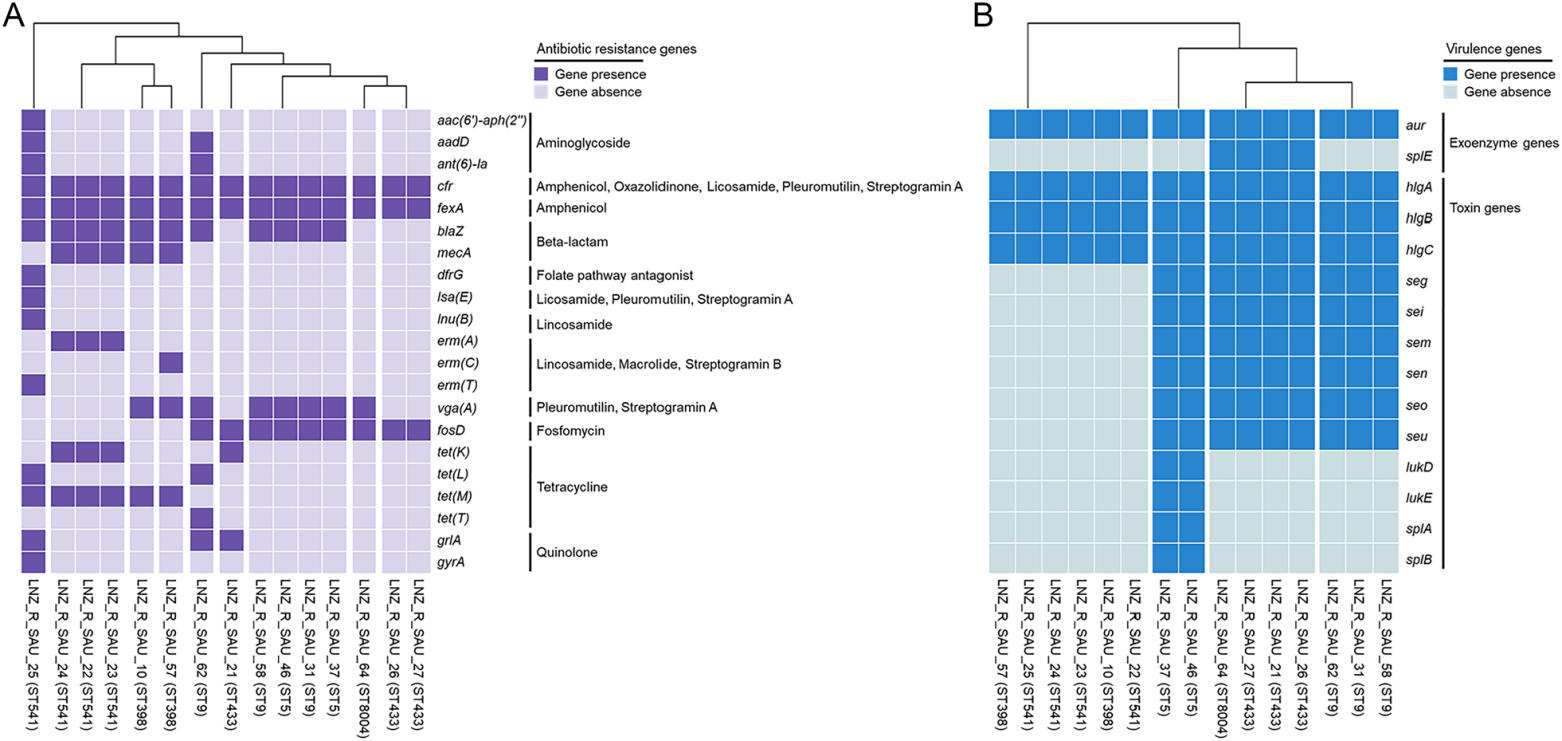
The heatmap of (**A**) antibiotic resistance gene and (**B**) virulence gene profiles across 15 *S*. *aureus* strains harboring *cfr* and *fexA* genes isolated in our previous study. Dark blocks represent the presence of genes and bright blocks represent absence. The labels on the bottom specify the tested isolates. Each row denotes one antibiotic resistance or virulence gene. The tree on the upper displays the relatedness of the isolates according to their antibiotic resistance and virulence profiles.

The genome sequences of *S. aureus* were analyzed for genetic features associated with virulence and drug resistance. The toxin gene distribution was associated with the ST type. Both livestock- and human-associated *S*. *aureus* strains carried enterotoxins (ST433, ST9, and ST5). In addition, two ST5 *S. aureus* strains carried leukocidin genes (*lukD* and *lukE*). However, livestock-associated *S. aureus* only carried hemolysin genes (*hlgA*, *hlgB*, and *hlgC*). Fifteen virulence genes were identified among the 15 strains sequenced in this study (Fig. 3). A heatmap was drawn to visualize the presence or absence of virulence genes. *aur*, *hlgA*, *hlgB*, and *hlgC* were the most widespread genes, being detected in all 15 strains.

### Detection of antibiotic resistance factors

In general, livestock-associated *S. aureus* carried a wider variety of resistance genes than human-associated *S. aureus*. In particular, livestock-associated *S. aureus* was resistant to antibiotics commonly used in veterinary medicine, and these strains harbored the corresponding resistance genes, such as tetracycline [*tet(K)*, *tet(L)*, *tet(M)*, and *tet(T)*] and macrolide resistance genes (*ermA*, *ermC*, *ermT*). In this study, 21 antibiotic resistance genes were identified among the 15 genomes sequenced in this study. These genes encode resistance to aminoglycoside [*aac(6′)-aph(2′′)*, *aadD*, and *ant(6)-Ia*], amphenicol (*fexA* and *cfr*), β-lactams (*blaZ* and *mecA*), folate pathway antagonists [*dfr(G)*], fosfomycin (*fosD*), lincosamides [*cfr*, *lnu(B)*, *lsa(E)*, *erm(A)*, *erm(C)*, and *erm(T)*], macrolides [*erm(A)*, *erm(C)*, and *erm(T)*], oxazolidinone (*cfr*), pleuromutilin [*cfr*, *vga(A)*, and *Isa(E)*], quinolone (*grlA* and *gyrA*), streptogramins [*cfr*, *vga(A)*, *Isa(E)*, *erm(A)*, *erm(C)*, and *erm(T)*], and tetracycline [*tet(K)*, *tet(L)*, *tet(M)*, and *tet(T)*; Fig. 3]. Among the 15 strains, five (33.3%) were identified as MRSA carrying *mecA*. The sequenced *S. aureus* strains featured nine resistance patterns, all of which were MDR. The LNZ_R_SAU_25 strain exhibited an MDR gene profile with the highest number of antibiotic resistance genes (n = 14) comprising 11 antibiotic classes. The most common gene profile was *cfr*, *fexA*, *blaZ*, *fosD*, and *vga(A)*, being found in four strains (26.7%), followed by *cfr*, *fexA*, *blaZ*, *mecA*, *erm(A)*, *tet(K)*, and *tet(M)*, which was present in three strains (20%).

### Mobile genetic elements

Mobile genetic elements, such as genomic islands, plasmid replicons, insertion sequences, and transposons, in the 15 strains were identified using the MobileElementFinder and IslandViewer databases (Additional file 3). Regarding transposons, *Tn6009* and/or *Tn554* were identified in nine *S. aureus* strains, whereas the remaining six strains carried no transposons. The insertion sequences *ISSau9*, *ISSau8*, *IS256*, *ISSau3*, and/or *ISSau1* and the plasmid replicon sequences *repUS43*, *repUS5*, *repUS18*, *rep7a*, *rep22*, *rep21*, *rep10*, *rep10b*, and/or *repUS70* were found in all strains. Between 2 and 14 genomic islands in each strain were predicted by IslandViewer. In all strains, *cfr* and *fexA* were located in genomic islands. Genomic islands harboring these genes varied in length from 12,151 to 140,021 bp. Regarding virulence factors, genomic islands harboring enterotoxin gene clusters were found in nine strains (LNZ_R_SAU_21, LNZ_R_SAU_26, LNZ_R_SAU_28, LNZ_R_SAU_31, LNZ_R_SAU_37, LNZ_R_SAU_46, LNZ_R_SAU_58, LNZ_R_SAU_62, and LNZ_R_SAU_64). These genomic islands differed from those carrying *cfr* and *fexA*. The length of genomic islands carrying enterotoxin gene clusters ranged 4275–8603 bp.

### Genetic environment of *cfr* and *fexA*

**Fig. 4:**
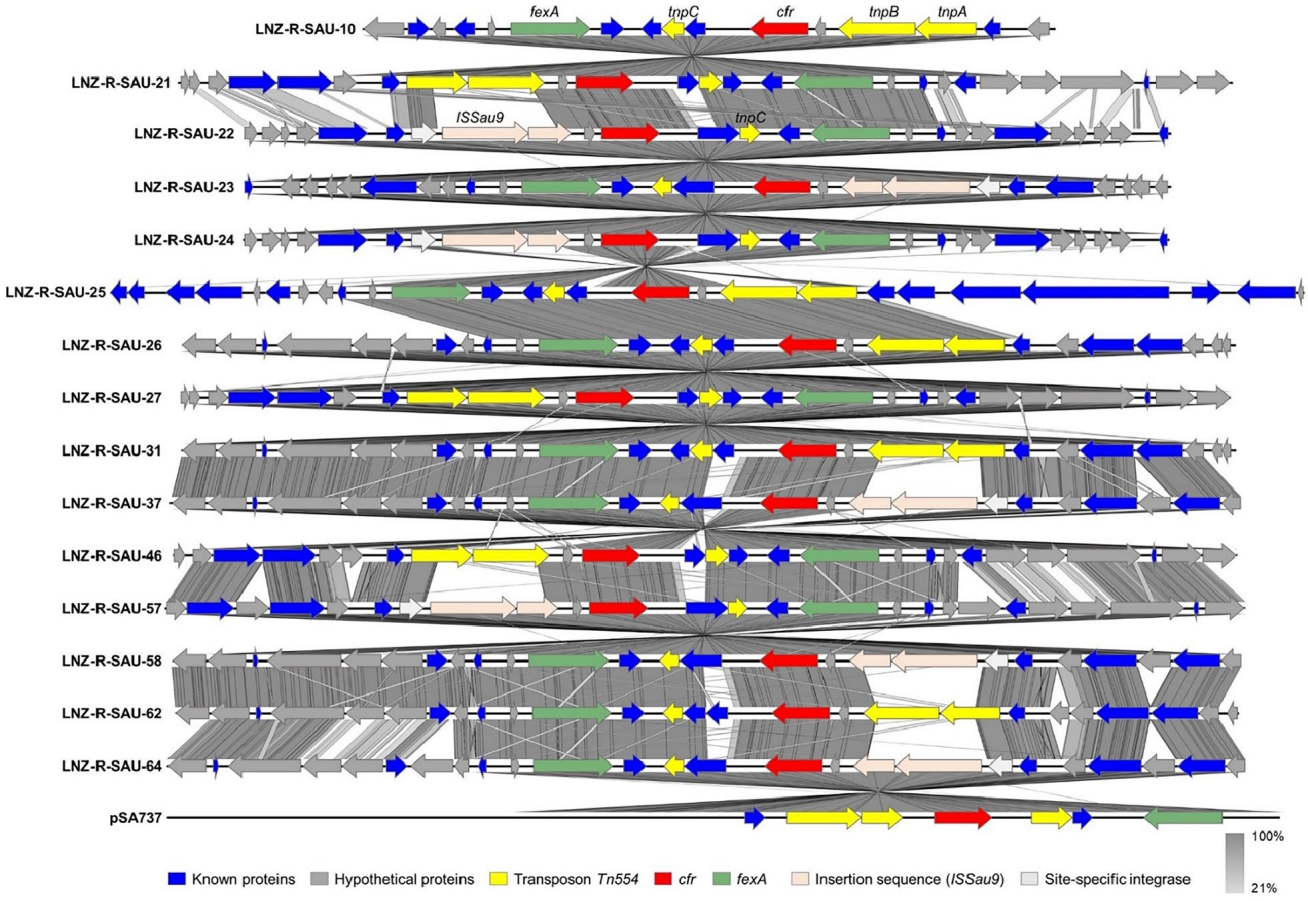
Schematic representation of the genetic environment of *cfr* gene in 15 *S*. *aureus* strains and *S. aureus* strain 004-737X plasmid pSA737 (accession no. KC206006.1) harboring *cfr* and *fexA* genes. Gene orientation is shown with arrows. The *cfr* and *fexA* genes are shown in red and green colored arrows. Grey lines connect regions with > 21% identity, and dark color indicates a higher percentage of identity.

**Fig. 5:**
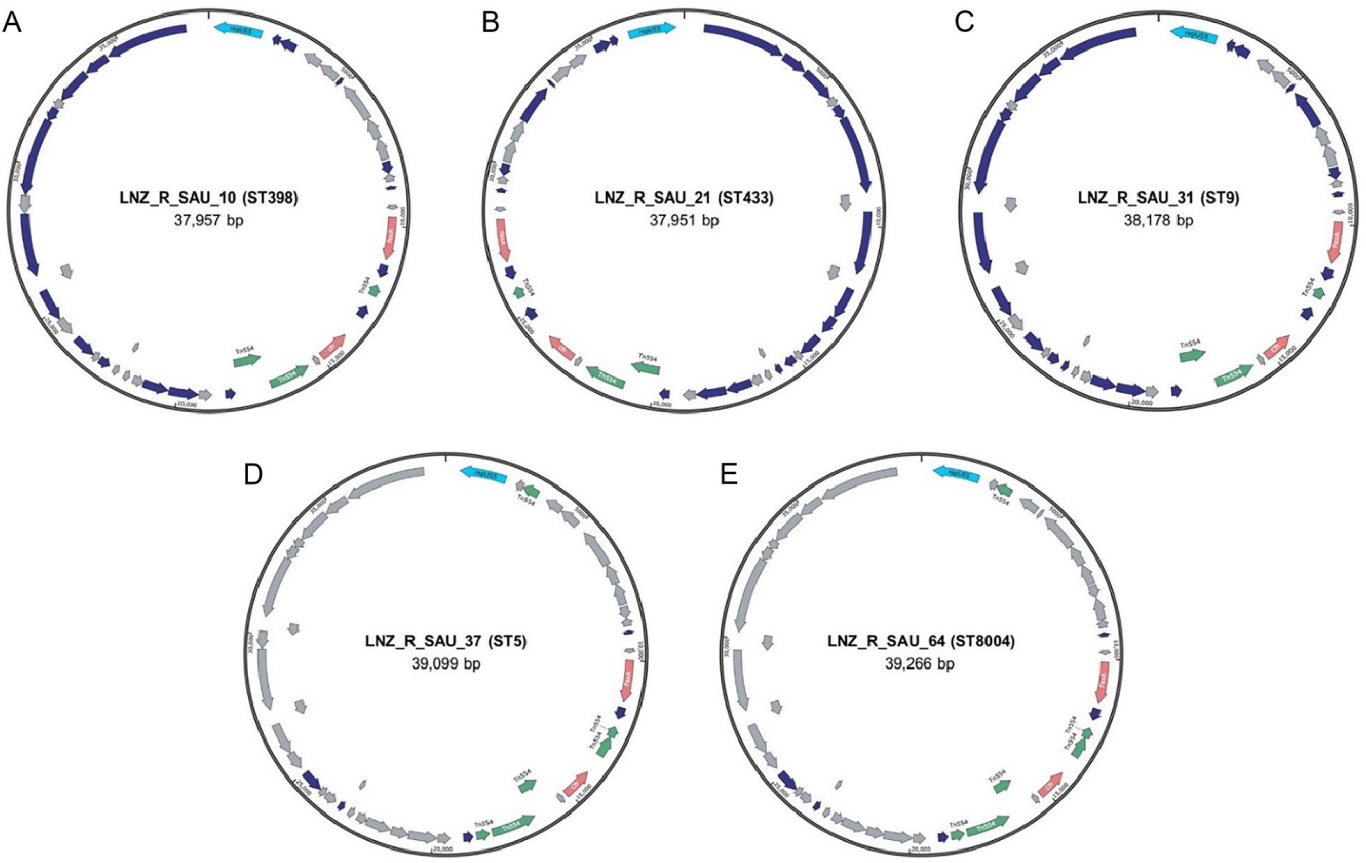
Plasmid map of *cfr* and *fexA* genes-containing plasmids of (**A**) LNZ_R_SAU_10, (**B**) LNZ_R_SAU_21, (**C**) LNZ_R_SAU_31, (**D**) LNZ_R_SAU_37, and (**E**) LNZ_R_SAU_64. Gene and their orientation are indicated by arrows as follows: red, green, blue, purple, and gray represent antibiotic resistance genes, IS elements, plasmid replicon, other proteins, and hypothetical proteins, respectively.

All 15 strains sequenced in this study harbored *cfr* and *fexA*. In all strains, *fexA* was located 2668 or 2818 bp upstream of *cfr* (Fig. 4). *Tn554* contains three transposition genes named *tnpA*, *tnpB*, and *tnpC* [26]. *tnpA* and *tnpB* of transposon *Tn554* were detected downstream of *cfr* in the LNZ_R_SAU_10, LNZ_R_SAU_25, LNZ_R_SAU_26, LNZ_R_SAU_31, and LNZ_R_SAU_62 strains, whereas *tnpC* was detected upstream of *cfr*. In the remaining strains, *ISSau9* was detected downstream of *cfr*, whereas *tnpC* was detected upstream. Analysis of the flaking region of *cfr* revealed that this gene was located around the plasmid replicon *repUS5* in 11 strains (LNZ_R_SAU_10, LNZ_R_SAU_21, LNZ_R_SAU_26, LNZ_R_SAU_27, LNZ_R_SAU_31, LNZ_R_SAU_37, LNZ_R_SAU_46, LNZ_R_SAU_57, LNZ_R_SAU_58, LNZ_R_SAU_62, and LNZ_R_SAU_64; Fig. 5 and Additional file 4), indicating its potential for transmission. Among them, 10 strains harbored sequences highly similar to that of the *S. aureus* strain 004–737 X plasmid pSA737 (accession no. KC206006.1, identity 99%, query coverage 84–100%), whereas the sequence of the LNZ_R_SAU_57 strain had high identity to that of the *S. aureus* strain 359 plasmid unnamed2 (accession no. CP077935.1, identity 100%, query coverage 97%). The LNZ_R_SAU_22, LNZ_R_SAU_23, LNZ_R_SAU_24, and LNZ_R_SAU_25 strains did not feature a replicon flanking *cfr*.

## Discussion

Linezolid is an antibiotic of last resort against highly resistant and complicated *S. aureus* infections in humans [27]. Although it is not approved for use in veterinary medicine, linezolid-resistant staphylococcal strains have been found on livestock farms and in foods [15, 21, 28–31]. The emergence of linezolid-resistant staphylococci poses a major threat to human and animal health because of the possibility of horizontal gene transfer between animals and humans and through direct contact or the food chain [27]. Consequently, linezolid-resistant staphylococci can be transmitted to humans through contact with livestock or food-producing animals, and infections caused by linezolid-resistant staphylococci could be difficult to treat [14]. The emergence of *cfr*-like gene-mediated linezolid resistance in staphylococci from humans and livestock has also been reported in clinical isolates in the United States, pigs and humans in Belgium, pigs in Korea, and turkeys in Egypt [15, 28, 32, 33]. Moreover, MRSA strains positive for *cfr*-like genes [*cfr*, *cfr(B)*, *cfr(C)*, *cfr(D)*, and *cfr(E)*] have been reported sporadically in livestock in several countries, such as Portugal, Germany, and Taiwan, and in hospitalized patients in China [14, 27, 31, 33]. Interestingly, linezolid-resistant staphylococci were recently detected in pig carcasses in Korea. A previous study found that 2.3% of more than 2500 *S. aureus* strains were resistant to linezolid [21]. These findings highlight the urgency of monitoring linezolid resistance in gram-positive pathogenic bacteria, including staphylococci, isolated from animals in Korea.

In this study, *cfr*- and *fexA*-carrying *S. aureus* obtained from pig carcasses at slaughterhouses in five provinces in Korea were analyzed in detail by WGS (Additional file 1) and compared with other genomes. The 111 publicly available genomes isolated from pig-related sources were associated with eight MLST types, and the most frequent allele profiles were ST398 (55 strains), ST5 (23 strains), and ST188 (10 strains). The predominant prevalence of genomes of ST398 strains in the database might be attributable to the scope of the studies in which these strains were obtained and sequenced, such as livestock-associated MRSA studies. The 15 strains were associated with six MLST types, indicating high diversity and complexity regarding their genomic backgrounds. Five of these sequence types (ST398, ST541, ST433, ST9, and ST8004) are livestock-associated *S*. *aureus* sequence types, whereas ST5 is the only human-associated *S*. *aureus* sequence type [21, 34]. ST9 is associated with pigs and human workers on livestock-related farms. Excluding ST8004, all *S*. *aureus* lineages belong to these MLST types, suggesting the possibility of transmission between livestock and humans. Among all strains, five MRSA strains belonging to sequence types ST398 and ST541 were methicillin-resistant. In particular, three strains belonged to ST541, a livestock-associated MRSA clone in Korea, and the remaining two strains were classified as ST398 [35, 36]. ST433 is known as linezolid-resistant MRSA carrying enterotoxin genes with host specificity [37, 38]. However, although ST433 harbored enterotoxin genes, it was not identified as MRSA in this study. In previous studies, most MRSA isolates from Asia were categorized as ST9, whereas most European strains were categorized as ST398 [33, 39]. Recently, the emergence of ST398 in pigs was reported in China and Japan, illustrating the possibility of transmission of the clone from livestock to humans [31, 40, 41]. Moreover, MRSA ST398 strains were detected in pigs or humans living close to pig farms in previous studies [27, 28, 40]. The MRSA ST398 strains isolated in this study harbored genes conferring methicillin and phenol resistance flanked by mobile genetic elements. Strains belonging to ST398 can be transmitted via livestock, consistent with previous reports describing the potential of ST398 to be transmitted from animal reservoirs to humans [31].

The ability of *S. aureus* to infect humans and animals is attributable to its arsenal of virulence factors, such as genes encoding proteins for tissue attachment, enzyme-degrading proteins, and leukocidins [42]. The genomes of *S*. *aureus* were sequenced and analyzed to identify known virulence factors. In total, 15 virulence genes were observed among the 15 strains. *aur*, *hlgA*, *hlgB*, and *hlgC* are highly widespread virulence factors within *S*. *aureus* genomes [11, 42], and these genes were present in all strains in this study. However, two strains (LNZ_R_SAU_37 and LNZ_R_SAU_46) harbored *lukD*/*lukE* related to leukotoxins and *splA*/*splB* encoding serine protease-like proteins [43, 44]. Nine strains harbored an enterotoxin gene cluster (*seg*, *sei*, *sem*, *sen*, *seo*, or *seu*) causing foodborne outbreaks [45]. Previous studies revealed that the enterotoxin gene cluster in *S*. *aureus* is located in plasmids or genomic islands [42]. Consistent with previous studies, enterotoxin gene clusters were found on genomic islands in nine strains. These genomic islands did not contain antibiotic resistance genes. This suggests that enterotoxin gene clusters on genomic islands can be transmitted between bacteria via horizontal transfer. Another study reported that human-associated ST5 strains are more virulent than other strains [46]. In this study, ST5 strains (LNZ_R_SAU_37 and LNZ_R_SAU_46) carried the highest number of most virulence genes. These strains, which harbor leukotoxin and enterotoxin, can spread to humans through the food chain and cause illness.

Linezolid resistance is associated with mutations in the 23S rRNA gene and ribosomal proteins and/or the acquisition of *cfr*, *optrA*, and *poxtA*, which are carried in mobile genetic elements [14]. Accordingly, several studies detected *optrA*, *poxtA*, and *cfr* in linezolid-resistant *S. aureus* and *Enterococcus faecalis* strains from livestock and food [14, 15, 35]. In this study, linezolid-resistant, methicillin-sensitive *S. aureus* linezolid-resistant MRSA strains harbored *cfr*. In addition, these strains carried *fexA*. Both *cfr* and *fexA* were closely co-localized within the contig. Among the 111 publicly available genomes, 10 carried *cfr*, and all but one belonged to ST398. Conversely, *optrA* and *poxtA*, which can also mediate linezolid resistance and which are often present in mobile genetic elements, were not identified in this study. This is likely because these genes are typically observed in enterococci rather than staphylococci. All *S. aureus* strains carrying *cfr* and *fexA* exhibited rather strong linezolid (MIC ≥ 8 mg/L) and chloramphenicol resistance (MIC > 64 mg/L) [21]. The frequent use of phenols and pleuromutilins on Korean livestock farms could be associated with the co-selection of linezolid resistance [21].

*S. aureus* is considered a reservoir of antibiotic resistance genes, and insertion sequences or transposons play an important role in the propagation of genes, requiring monitoring to detect transferable antibiotic-resistant strains [32]. The *repUS5* was found in all strains, excluding four ST541 strains. The *repUS5* was previously found in *S. aureus* isolated from poultry, and it has been linked to antimicrobial resistance gene transfer in staphylococci [47]. BLAST analysis revealed that the *cfr* and *fexA*-carrying fragments had high similarities with a plasmid of the *S. aureus* strain 004-737X (pSA737) isolated from a clinical sample in the United States and a plasmid of *S. aureus* strain 359 (unnamed2) isolated from human in Germany [48]. Meanwhile, *cfr* was flanked by *Tn554*-related *tnpA*, *tnpB*, and *tnpC* or *ISSau9* (also called IS21-558) in all strains. Among the 111 publicly available genomes, 10 harbored *cfr*. These strains were isolated from pig-related sources in Australia (one strain), the Netherlands (one strain), China (three strains), and Belgium (five strains). Consistent with our findings, *cfr* was flanked by *Tn554*-related *tnpA*, *tnpB*, and *tnpC* or *ISSau9* in these strains (Additional file 5). In previous studies, *Tn554*-mediated *optrA* transfer was detected in the chromosome carrying *optrA*, indicating that this gene can be transmitted between bacterial species [49, 50]. *ISSau9*, originally detected in the pSCFS3 plasmid recovered from an *S. aureus* strain of pig origin in Germany, was also found in the pGMI17-006 plasmid from a human *S*. *aureus* strain from Denmark [27, 51]. The *cfr* in these strains is flanked by *ISSau9* and *Tn554*. Therefore, *Tn554* and *ISSau9* could play important roles in the horizontal transmission of *cfr* and *fexA* in other pathogenic bacteria. These data indicate the need for continued surveillance of linezolid-resistant *S. aureus* carrying mobile genetic elements.

In the phylogenetic analysis, the LNZ_R_SAU_10 and LNZ_R_SAU_57 strains were grouped with *S*. *aureus* strains isolated from pigs in Belgium (Fig. 1). These strains belong to ST393, and they harbor *cfr* flanked by *Tn554*-related *tnpA*, *tnpB*, and *tnpC*. LNZ_R_SAU_31, LNZ_R_SAU_58, and LNZ_R_SAU_62 were grouped with *S*. *aureus* strains isolated from pig skin in the United States, and all of these strains belonged to ST9. LNZ_R_SAU_37 and LNZ_R_SAU_46 were grouped with the ISU 998 strain (sapig_32) isolated from a swine facility in the United States, and all of these strains belonged to ST5. LNZ_R_SAU_21, LNZ_R_SAU_26, and LNZ_R_SAU_27 strains were grouped with *S*. *aureus* strains isolated from a pig farm in Australia, and all of these strains belonged to ST433. Four strains (LNZ_R_SAU_22, LNZ_R_SAU_23, LNZ_R_SAU_24, and LNZ_R_SAU_25) belonging to ST541 were grouped with ST398 strains isolated from pig farm dust and pig farms in Italy and China. ST541 is a single-locus variant of ST398. Although ST541 and ST398 are closely related, they exhibit different antibiotic resistance patterns [27]. ST541 has been occasionally found in MRSA strains in Korea, but it has not yet been reported in other countries. Similarly, four linezolid-resistant *S. aureus* ST541 strains were detected in this study, and all but one (LNZ_R_SAU_25) harbored *mecA* conferring methicillin resistance. The LNZ_R_SAU_64 strain (ST8004) was grouped with the CFSAN018749 strain (ST433) isolated from tissue and/or biological fluid from swine in Denmark. This study detected the ST8004 strain (LNZ_R_SAU_64) in pig carcasses for the first time. This strain was resistant to linezolid (MIC = 8 mg/L), chloramphenicol (MIC = 64 mg/L), clindamycin (MIC > 4 mg/L), tiamulin (MIC > 4 mg/L), and quinupristin/dalfopristin (MIC > 4 mg/L) [21]. ST8004 (allele profile 2-2-637-2-6-3-72) is a single-locus variant of ST433 (allele profile 2-2-2-2-6-3-72). These two sequence types featured different antibiotic resistance genes. Specifically, all ST433 strains commonly carried *cfr*, *fexA*, and *fosD*, whereas the ST8004 isolate harbored *cfr*, *fexA*, *fosD*, and *vga(A)*. This difference in antibiotic resistance genes between extremely close types, such as ST433 and ST8004, could be attributable to the acquisition of mobile genetic elements.

This study highlighted that transferable linezolid resistance and virulence genes in *S. aureus* strains could persist in pig carcasses. Mobile genetic elements, such as plasmid replicons (*repUS5*), *Tn554*, and *ISSau9*, might mediate the horizontal transfer of *cfr* and *fexA* in *S. aureus* strains in pig carcasses, whereas genomic islands could play a similar role in the horizontal transfer of enterotoxin gene clusters. These results suggest that linezolid resistance and virulence genes in *S. aureus* strains have diverse transmission properties in livestock or food-producing animals. In addition, linezolid-resistant *S. aureus* can be transmitted from pigs to humans, and *S. aureus* in pigs used for food production could represent an important repository of transferable linezolid resistance and virulence genes. The prevalence and transmission of transferable genes in *S. aureus* strains from livestock or food-producing pigs should be continually monitored.

### Supplementary Information

**Additional file 1: **Genomic features of 106 *S. aureus* strains used in this study

**Additional file 2: **MLST analysis of *S. aureus* strains harboring *cfr(A)* gene.

**Additional file 3: **Mobile genetic elements in 15 *S. aureus* strains.

**Additional file 4: **Plasmid map of *cfr* and *fexA* genes-containing plasmids of (A) LNZ_R_SAU_26, (B) LNZ_R_SAU_27, (C) LNZ_R_SAU_46, (D) LNZ_R_SAU_57, (E) LNZ_R_SAU_58, and (F) LNZ_R_SAU_62. Gene and their orientation are indicated by arrows as follows: red, green, blue, purple, and gray represent antibiotic resistance genes, IS elements, plasmid replicon, other proteins, and hypothetical proteins, respectively.

**Additional file 5: **Schematic representation of the genetic environment of *cfr* and *fexA* genes in 10 publicly available genomes harbored *cfr* and *fexA* genes. Gene orientation are shown with arrows. The *cfr* and *fexA* genes are shown in red and green colored arrows. Grey lines connect regions with >20% identity, and dark color indicates a higher percentage of identity.

## Data Availability

The datasets are available from the corresponding author on reasonable request.
