# Supplementary figures and images for: Genomic characteristics of cfr and fexA carrying Staphylococcus aureus isolated from pig carcasses in Korea
Source: Vet Res. 2024 Feb 16;55:21. doi: 10.1186/s13567-024-01278-x (PMC10874063; doi:10.1186/s13567-024-01278-x)

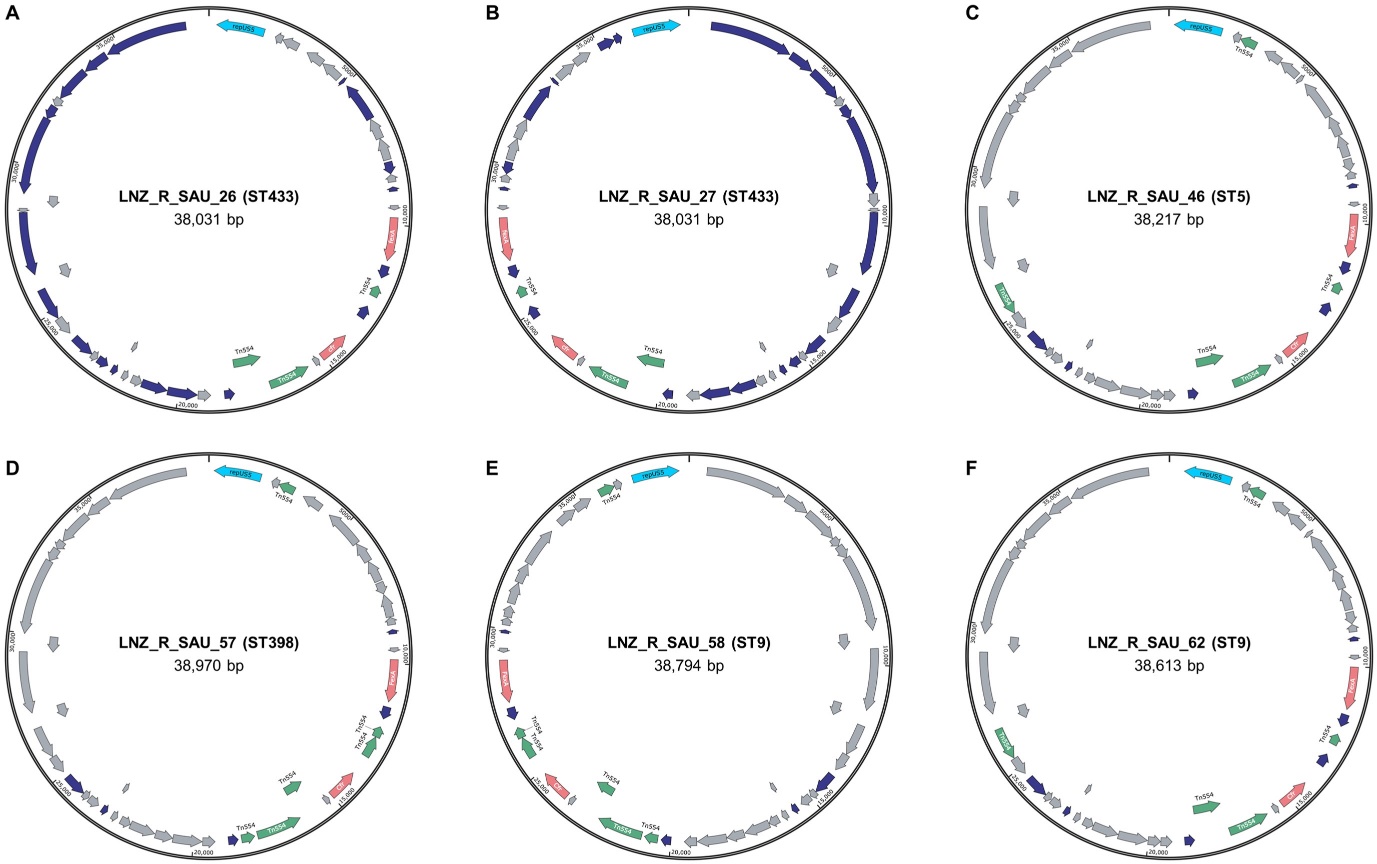

Supplement: Supplementary file 4 — Additional file 4: Plasmid map of cfr and fexA genes-containing plasmids of (A) LNZ_R_SAU_26, (B) LNZ_R_SAU_27, (C) LNZ_R_SAU_46, (D) LNZ_R_SAU_57, (E) LNZ_R_SAU_58, and (F) LNZ_R_SAU_62. Gene and their orientation are indicated by arrows as follows: red, green, blue, purple, and gray represent antibiotic resistance genes, IS elements, plasmid replicon, other proteins, and hypothetical proteins, respectively. [file 13567_2024_1278_MOESM4_ESM.docx]

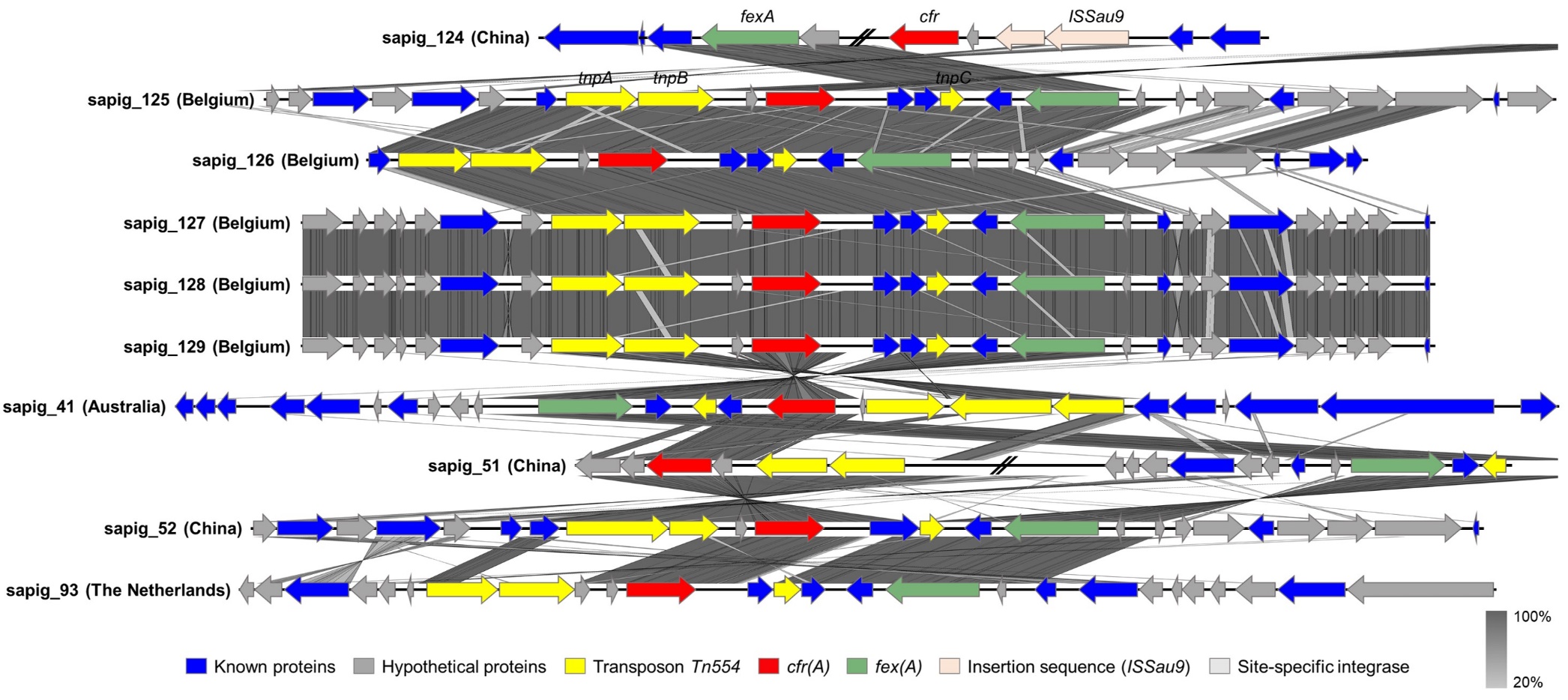

Supplement: Supplementary file 5 — Additional file 5: Schematic representation of the genetic environment of cfr and fexA genes in 10 publicly available genomes harbored cfr and fexA genes. Gene orientation are shown with arrows. The cfr and fexA genes are shown in red and green colored arrows. Grey lines connect regions with >20% identity, and dark color indicates a higher percentage of identity. [file 13567_2024_1278_MOESM5_ESM.docx]
